# Identifying biomarkers for breast cancer by gene regulatory network rewiring

**DOI:** 10.1186/s12859-021-04225-1

**Published:** 2022-01-20

**Authors:** Yijuan Wang, Zhi-Ping Liu

**Affiliations:** Department of Biomedical Engineering, School of Control Science and Engineering, Shandong University, Jinan, 250061 Shandong China

**Keywords:** Biomarker discovery, Gene regulatory network, Network rewiring, Feature selection, Breast cancer

## Abstract

**Background:**

Mining gene regulatory network (GRN) is an important avenue for addressing cancer mechanism. Mutations in cancer genome perturb GRN and cause a rewiring in an orchestrated network. Hence, the exploration of gene regulatory network rewiring is significant to discover potential biomarkers and indicators for discriminating cancer phenotypes.

**Results:**

Here, we propose a new bioinformatics method of identifying biomarkers based on network rewiring in different states. It firstly reconstructs GRN in different phenotypic conditions from gene expression data with a priori background network. We employ the algorithm based on path consistency algorithm and conditional mutual information to delete false-positive regulatory interactions between independent nodes/genes or not closely related gene pairs. And then a differential gene regulatory network (D-GRN) is constructed from the rewiring parts in the two phenotype-specific GRNs. Community detection technique is then applied for D-GRN to detect functional modules. Finally, we apply logistic regression classifier with recursive feature elimination to select biomarker genes in each module individually. The extracted feature genes result in a gene set of biomarkers with impressing ability to distinguish normal samples from controls. We verify the identified biomarkers in external independent validation datasets. For a proof-of-concept study, we apply the framework to identify diagnostic biomarkers of breast cancer. The identified biomarkers obtain a maximum AUC of 0.985 in the internal sample classification experiments. And these biomarkers achieve a maximum AUC of 0.989 in the external validations.

**Conclusion:**

In conclusion, network rewiring reveals significant differences between different phenotypes, which indicating cancer dysfunctional mechanisms. With the development of sequencing technology, the amount and quality of gene expression data become available. Condition-specific gene regulatory networks that are close to the real regulations in different states will be established. Revealing the network rewiring will greatly benefit the discovery of biomarkers or signatures for phenotypes. D-GRN is a general method to meet this demand of deciphering the high-throughput data for biomarker discovery. It is also easy to be extended for identifying biomarkers of other complex diseases beyond breast cancer.

## Background

Gene regulatory network (GRN) is a model that characterizes the complex relationship between genes in a cell [1]. In a GRN, nodes represent genes and edges describe the regulatory relationships among them. From a physical perspective, the interactions between genes are through their products like proteins and RNAs. The weight of edge describes the direction and strength of an interaction. The alternation or mutation of one gene may affect the activity of many other genes through the network [2, 3].

Cancer is recognized as a complex disease caused by gene mutations, which will perturb the normal interactions among genes and lead to the disorder of connection mode or strength [4–6]. In other words, gene mutations cause perturbation and rewiring of GRNs [7, 8]. The rewired interactions generate changes in normal biological processes and that is crucial for cancerogenesis. Thus, the investigation of the rewiring GRN is significant in discovering potential biomarkers of indicating certain phenotypic states.

Breast cancer is the most commonly diagnosed cancer and the second leading cause of cancer death in women worldwide [9, 10]. Biomarkers play important roles in its early diagnosis and prognostic evaluation [11–13]. Nowadays, the accurate identification of biomarkers for breast cancer early detection is still very challenging. There are some biomarkers that have been validated like BRCA1 and HER2 [14]. However, new biomarkers and their combinations are still urgently needed to quantify the treatment effects with classical clinical prognostic factors. They also indicate the potential risks and pathogenesis of breast cancer [15, 16].

With the development of high-throughput sequencing technologies, an increasing amount of gene expression data become available. Various methods have been developed to find efficient biomarkers from high-throughput data [17–20]. For instance, the methods construct a dynamic network model and perform a multi-omics data integration for biomarker discovery [21]. However, there are few methods to solve this problem from the perspective of network rewiring, which indicates the dysfunctional mechanism of cancer.

In this paper, we propose a framework to identify potential biomarkers of breast cancer based on network rewiring. The disease and normal GRN are reconstructed from gene expression data with a reliable background GRN. CMI-PC (conditional mutual information-based path consistency) algorithm is employed to delete false positive interactions between independent genes or pairs that are not connected closely in a specific condition from the integrative background network. Comparing the GRNs in the two phenotypic conditions, a differential GRN, called D-GRN, containing the rewired nodes with differential regulations will be extracted. In D-GRN, we detect the community structures which are intensively connected nodes in the form of subnetwork modules. Finally, we apply logistic regression with recursive feature elimination (LR-RFE) to select biomarkers in each module respectively. We use cross-validations to find the optimal number of biomarkers individually. The maximum AUC in these module-based biomarkers achieves 0.985 in the internal validation. The selected biomarkers are also verified in external independent datasets and they achieve the maximum AUC value of 0.989 in classification.

## Results

In this work, the proposed biomarker discovery framework focuses on the rewiring gene network between disease and normal conditions. Condition-specific GRNs are reconstructed through the integration of prior knowledge of an integrative background network and phenotypic gene expression data. CMI-PC algorithm is employed to remove redundant regulatory interactions from the background network. D-GRN is extracted from the two specified networks in two different states. We detect the communities in the D-GRN. And then machine learning method is applied to find the best feature combination in classification experiments. The selected features are more likely to be potential biomarkers. Here, we apply our framework to breast cancer and identify potential network-based module biomarkers.

### Network rewiring

The reconstructed normal GRN has 430 edges (regulations) and 198 nodes (genes), while the disease GRN has 301 edges and 137 nodes. There are 71 same genes and 115 common edges between them. We merge the same nodes that have different connections and their neighbors to construct a D-GRN which contains 509 regulations and 238 genes.

**Fig. 1:**
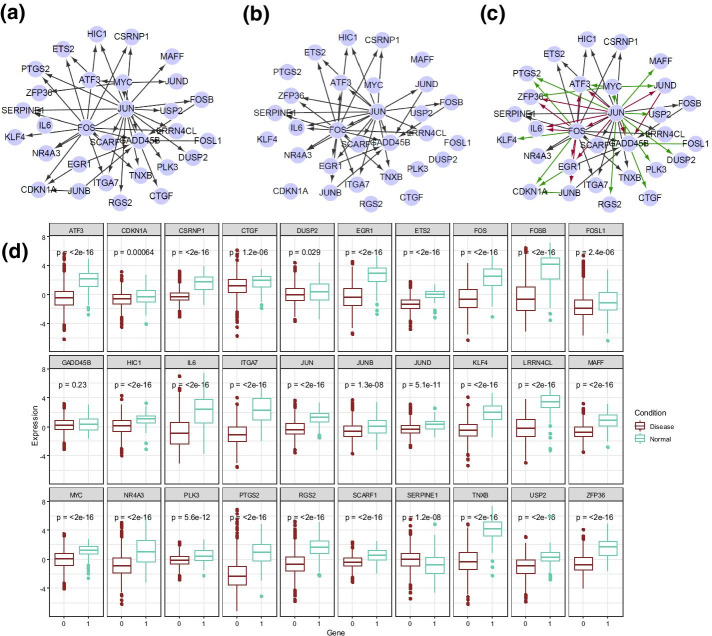
Network rewiring and gene expression analysis of an identified module (Module 4). **a** Regulatory interactions in normal condition. **b** Regulatory interactions in disease condition. **c** D-GRN. **d** Gene expression profiles in normal and disease

After community detection, the D-GRN has been divided into 5 modules (in the next section). To illustrate the network rewiring in normal and disease states, Fig. 1a, b show the Module 4’s gene regulatory interactions in normal condition and disease condition respectively. Figure 1c illustrates this part of D-GRN, including 30 nodes. Black, green and red lines represent edges in common, only in the normal network and only in the disease network respectively. Figure 1d shows the gene expression boxplot details in the normal and disease conditions and *P* values of difference. It can be easily observed that most nodes have significantly different gene expressions between the two conditions. Interestingly, few genes are not differentially expressed, but the regulatory interactions rewire in the two conditions. Instead of the node-centric difference, D-GRN identifies the edge-centric difference between the two phenotypes, i.e., normal and cancer state by the rewiring gene regulations.

**Fig. 2:**
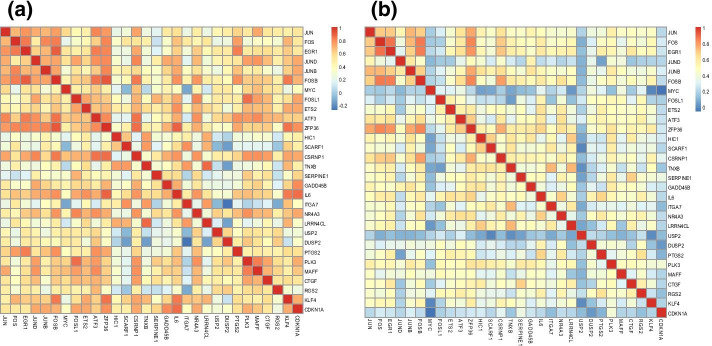
Correlation between genes in Module 4. **a** Normal condition. **b** Cancer condition

To further demonstrate the perturbations in the two GRNs, Fig. 2a, b present the heatmaps of Pearson’s correlation coefficient (PCC) between genes in normal and disease conditions. Obviously, there is a marked difference between them and it proves the effectiveness of our identification of the rewiring GRN across two conditions.

### Detected communities

**Fig. 3:**
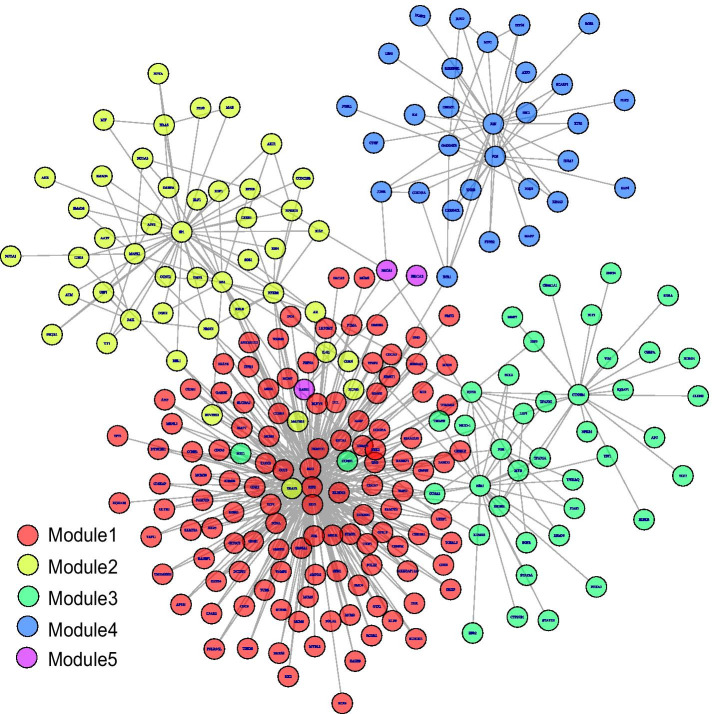
Community detection result in D-GRN

The community detection results in D-GRN are shown in Fig. 3. Different colors correspond to different modules. The 5 modules include 118, 46, 41, 30, and 3 members of genes individually. The global D-GRN is then divided into five functional blocks in the form of network-based modules. These subnetworks provide a pool of module biomarker candidates. To remove the redundant genes in the five detected modules, we perform feature selection for discovering biomarker gene sets respectively.

### Breast cancer biomarkers identification and validation

**Fig. 4:**
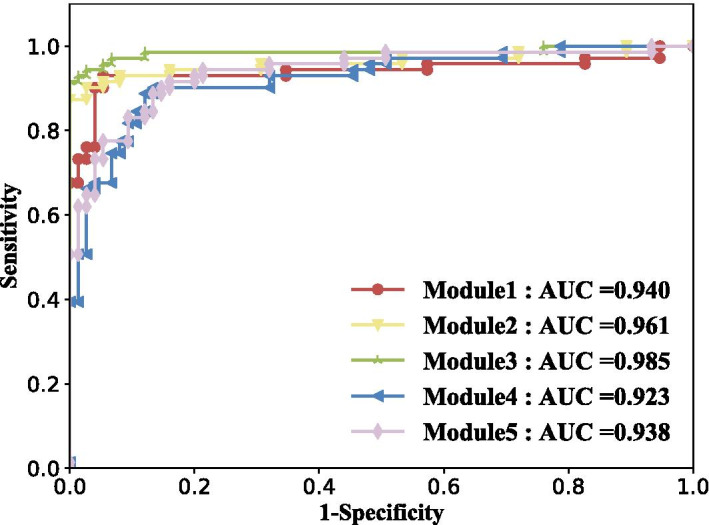
Classification performance of identified biomarkers in internal validation data

Table 1 lists the selected genes in each module after logistic regression with recursive feature elimination (LR-REF) with tenfold cross-validations. F1-scores in classification experiments are also shown. Due to the number of genes in each module is diverse and some specific genes may have better discrimination abilities, there is a fluctuation of F1-score in the five modules. However, all of them are over 0.86, which means they perform well in the classification of distinguishing disease samples from controls. Figure 4 shows the receiver operating characteristic (ROC) curves of the selected biomarkers underlying the five modules in the internal validation dataset. The highest area under the ROC curve (AUC) value achieves 0.985, and the lowest reaches 0.923.

**Table 1:** Five module biomarkers after LR-RFE selection

Module	Biomarker genes	F1-score
1	KLF9 UHRF1 CDC25A CCNE1 CDK2 CCNE2 TUBB TAF11 POLE2 PKMYT1 KLHDC1 CDC45 ZBTB4 UBE2S CDKN2C NEK2 TOMM40 TACC3 GPR19 TCEAL5 FUS SIK2 AP1S1 SHB HS6ST1 TP73 GATA3 HOXA10 CD3EAP SLC20A1 XKR5 SOX4	0.93
2	NFKB2 NFYA NR3C1 MAZ BAX TNIP2 DGKZ PIK3R1 IL4I1	0.92
3	KDM4B STAT5B BCL2 TRIM59	0.96
4	TNXB MAFF CTGF KLF4 JUN	0.86
5	BRCA1 RAD51	0.86

**Fig. 5:**
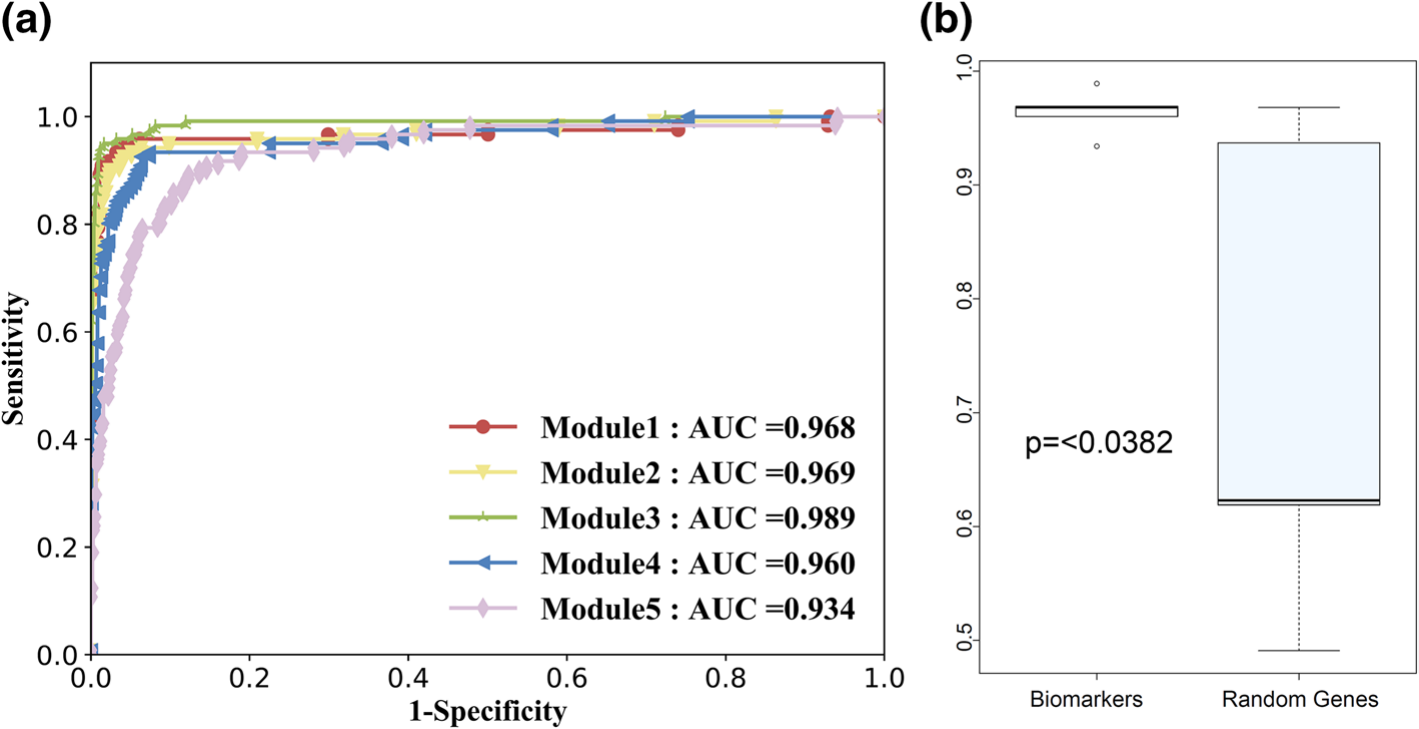
Classification performance of identified biomarkers and random genes in the independent validation data. **a** ROC curves of module biomarkers. **b** Comparison of the classification ability between module biomarkers and equal amount of random genes

For validating of our finding module biomarkers, we perform the classification experiments in the external independent dataset GSE42568, which contains 104 disease samples and 17 controls. Figure 5a demonstrates the ROC curves of each selected gene sets in the independent validation data. As shown, the 5 gene modules all perform well in the classifications. The highest AUC value achieves 0.989, and the lowest AUC value reaches 0.934. In addition, Fig. 5b shows the diverse ability of classification in module biomarkers and in the corresponding random gene sets with the same size. The *P*-value is 0.0382, indicating a significant difference for them in classification. The results provide evidence that they are potential molecular markers for diagnosing breast cancer.

To further demonstrate the dysfunctions of selected biomarkers, we employ network ontology analysis (NOA) [22] to perform gene ontology (GO) enrichment analysis on the rewired regulatory network across normal and disease states. Table 2 lists the enriched functions in the discovered biomarkers. As shown, some important cancerous dysregulations related ‘metabolic process’, ‘cell cycle’, ‘cell proliferation’ and ‘lymphocyte differentiation’ are significantly enriched. They are consistent with the prior knowledge of breast cancer pathogenesis during occurrence and development [23]. In turn, the functional analysis also provides evidence for the effectiveness of our proposed biomarker discovery method via network rewiring.

**Table 2:** The enriched GO biological processes of identified biomarkers in D-GRN

GO term	Description	Adjusted P-value	Biomarker
GO:0031323	Regulation of cellular metabolic process	1.90E−30	NFKB2, DGKZ, JUN, KLF9, RAD51, UHRF1, CDC25A, CCNE1, CDK2, CCNE2, TAF11, PKMYT1, ZBTB4, UBE2S, CDKN2C, KDM4B, BCL2, TCEAL5, NFYA, NR3C1, MAZ, MAFF, CTGF, KLF4, STAT5B, TP73, GATA3, HOXA10, SOX4, BRCA1
GO:0060255	Regulation of macromolecule metabolic process	7.40E−29	NFKB2, BAX, JUN, KLF9, RAD51, UHRF1, CCNE1, CDK2, TAF11, ZBTB4, UBE2S, KDM4B, BCL2, TCEAL5, NFYA, NR3C1, MAZ, MAFF, CTGF, KLF4, STAT5B, TP73, GATA3, HOXA10, SOX4, BRCA1
GO:0051171	Regulation of nitrogen compound metabolic process	4.00E−26	NFKB2, JUN, KLF9, RAD51, UHRF1, CCNE1, CDK2, TAF11, ZBTB4, KDM4B, TCEAL5, NFYA, NR3C1, MAZ, MAFF, KLF4, STAT5B, TP73, GATA3, HOXA10, SOX4, BRCA1
GO:0051726	Regulation of cell cycle	3.30E−24	DGKZ, JUN, CDC25A, CDK2, CCNE2, PKMYT1, CDKN2C, NEK2, TACC3, BCL2, CTGF, STAT5B, TP73, BRCA1
GO:0019219	Regulation of nucleobase, nucleoside, nucleotide and nucleic acid metabolic process	8.80E−24	NFKB2, JUN, KLF9, RAD51, UHRF1, CCNE1, CDK2, TAF11, ZBTB4, KDM4B, TCEAL5, NFYA, NR3C1, MAZ, MAFF, KLF4, STAT5B, TP73, GATA3, HOXA10, SOX4, BRCA1
GO:0010604	Positive Regulation of macromolecule metabolic process	1.10E−23	JUN, RAD51, CCNE1, CDK2, TAF11, UBE2S, BCL2, NFYA, CTGF, STAT5B, TP73, SOX4, BRCA1
GO:0051173	Positive regulation of nitrogen compound metabolic process	2.10E−23	JUN, RAD51, CCNE1, CDK2, TAF11, NFYA, STAT5B, TP73, SOX4, BRCA1
GO:0009893	Positive regulation of metabolic process	2.40E−23	JUN, RAD51, CCNE1, CDK2, TAF11, UBE2S, BCL2, NFYA, CTGF, STAT5B, TP73, SOX4, BRCA1
GO:0006357	Regulation of Transcription from RNA polymerase II promoter	1.00E−16	JUN, KLF9, UHRF1, NFYA, STAT5B, BRCA1
GO:0042127	Regulation of cell proliferation	1.20E−16	JUN, CDK2, CDKN2C, BCL2, CTGF, KLF4, STAT5B, SOX4, BRCA1
GO:0048545	Response to steroid hormone stimulus	5.60E−15	CCNE1, BCL2, CTGF, STAT5B, GATA3, BRCA1
GO:0051716	Cellular response to stimulus	1.70E−11	DGKZ, JUN, RAD51, UHRF1, CCNE1, POLE2, BCL2, PIK3R1, STAT5B, TP73, BRCA1
GO:0010941	Regulation of cell death	5.10E−10	BAX, JUN, TUBB, CDKN2C, BCL2, CTGF, STAT5B, TP73, SOX4, BRCA1
GO:0043067	Regulation of programmed cell death	1.70E−09	BAX, JUN, TUBB, CDKN2C, BCL2, CTGF, STAT5B, TP73, SOX4, BRCA1
GO:0042325	Regulation of Phosphorylation	6.80E−09	DGKZ, JUN, CDC25A, CCNE2, PKMYT1, CDKN2C, BCL2, CTGF, TP73
GO:0007346	Regulation of mitotic cell cycle	7.00E−09	DGKZ, CDK2, PKMYT1, NEK2, BCL2, STAT5B
GO:0051094	Positive regulation of developmental process	1.20E−08	BAX, JUN, CCNE1, BCL2, STAT5B
GO:0006974	Response to DNA damage stimulus	5.20E−07	DGKZ, RAD51, UHRF1, POLE2, TP73, BRCA1
GO:0000075	Cell cycle checkpoint	2.00E−06	DGKZ, CCNE2, BRCA1
GO:0045786	Negative regulation of cell cycle	2.60E−06	DGKZ, CDKN2C, BCL2, TP73
GO:0030522	Intracellular receptor mediated signaling pathway	2.90E−06	KLF9, CCNE1, BRCA1
GO:0048729	Tissue morphogenesis	4.00E−05	BCL2
GO:0030217	T cell differentiation	8.10E−05	BCL2, STAT5B, SOX4
GO:0002009	Morphogenesis of an epithelium	1.50E−04	BCL2
GO:0030098	Lymphocyte differentiation	8.90E−04	BCL2, STAT5B, SOX4

## Discussion

Identification of biomarkers for complex diseases such as cancer is of paramount importance in treatment, diagnosis and prognosis. Although numerous methods have been proposed to characterize biomarkers, few are from the perspective of regulatory network rewiring. GRN is one important strategy for revealing the disease mechanism from a systematic perspective. The investigation of cancer mutation and perturbation through GRN rewiring is of significance for addressing the underlying causal regulations responding to phenotypic transition. In this paper, we proposed a novel framework for identifying biomarkers based on network rewiring. Disease and normal condition-specific GRNs have been reconstructed from gene expression data with a priori background network respectively. The gene regulatory interactions changed between them illustrated the results of disease mutation and perturbation. D-GRN is extracted and modules in it are detected sequentially. LR-RFE is employed to find diagnostic biomarkers from modules. And cross-validation is used to set optimal number of biomarkers in each module.

Here, we applied the proposed framework D-GRN for identifying biomarkers of breast cancer. The integrative background network based on prior knowledge and condition-specific gene expression data have been used to construct normal and disease GRNs. We have to admit that there is limitation on missing nodes and edges, which is also expected to be as complete as possible. Totally, a D-GRN including 509 edges and 238 nodes have been extracted. Five potential biomarker gene sets in the form of subnetwork modules have been identified and they performed well in the classification of disease/normal samples in both internal and external validation datasets.

The focus of this work is to provide a computational pipeline for cancer biomarker discovery. In our framework, we select optimal genes serving as biomarkers in the network modules by machine learning. The rewired regulations as well as the weights or coefficients on these regulations have not been fully considered in biomarker discovery. The rewiring edges and patterns are expected to be embedded in the future discovery of biomarkers. In this work, another potential limitation is that the rewiring mechanism and gene dysfunction across different phenotypes have not been included in our feature selection. The genetic and epigenetic factors need be integrated together for addressing the causality of these identified gene regulatory rewiring. These will provide more valuable information for detecting more precise biomarkers for breast cancer.

## Conclusion

In conclusion, network rewiring reveals significant information about cancer mechanisms. With the development of high-throughput technology, the amount of high quality gene expression data will keep arising. Condition-specific networks that are close to the real gene network will be established. The rewiring network components will be more clearly revealed, which will greatly benefit the discovery of biomarkers or signatures for breast cancer diagnosis. Obviously, our proposed strategy is rather general and it can be used to discovering biomarkers for other complex diseases.

## Methods

### Data sources and pre-processing

The RNA-seq gene expression data are downloaded from the TCGA data portal that includes 1097 patients with BRCA (breast invasive carcinoma) and 112 normal controls. The dataset provides gene expression values in the form of mean-centered number for 17,924 genes in all samples. In this study, 60% samples are used for training and testing purpose. We call them as internal training datasets. The remaining 40% samples are used for internal validation. We also download an independent dataset from NCBI GEO database (ID: GSE42568) for validating the identified biomarkers. It has 104 cancer samples and 17 controls. They are called external independent validation data.

The integrative human GRN is downloaded from our RegNetwork knowledgebase [24]. RegNetwork is a comprehensive repository for GRN by collecting the documented gene regulations from more than 20 databases and the predicted gene regulations by aligning transcription factor binding sites. Here, we use a new version of it containing 151,215 regulations in 19,719 genes.

### Framework

**Fig. 6:**
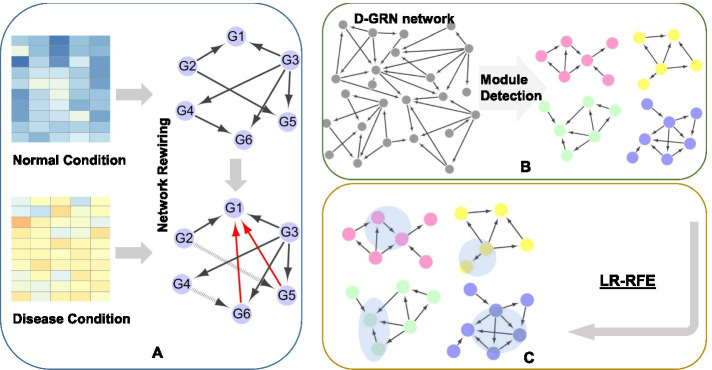
The framework of biomarker discovery based on network rewiring. **a** Reconstruct the disease and normal GRNs respectively by integrating prior background network and gene expression data. **b** Extract the rewiring regulations and establish a D-GRN. Module detection is implemented to find closely-connected nodes in D-GRN. **c** Identify biomarkers in each module through LR-RFE

Figure 6 shows the framework of biomarker identification. It mainly contains three steps. First, as shown in Fig. 6a, it acquires the background of GRN through our prior knowledge about gene regulations in humans. It is a non-specific regulatory network with many redundant gene regulations. Gene expression data in normal and disease samples are used to evaluate the prior gene–gene interactions in specific phenotypes and eliminate redundant ones in the background GRN. Second, by comparing the normal and disease specific GRNs reconstructed from gene expression data, we can clearly identify the rewiring network sections across the two phenotypic states. A differential GRN called D-GRN can be extracted by comparing them. Community detection algorithm is then employed to find closely-connected nodes in the form of modules as shown in Fig. 6b. Third, we apply a logistic regression with recursive feature elimination (LR-RFE) approach to find biomarker genes as shown in Fig. 6c.

### Gene regulatory network rewiring

Determining GRN is an important avenue for revealing disease mechanisms. In this study, disease GRN and normal GRN are reconstructed respectively based on the corresponding gene expression profiling data on a background network. The prior network is deposited in RegNetwork, a knowledge-based genome-wide regulatory network database by integrating amount of data resources [24].

Numerous methods have been developed to reconstruct GRN from gene expression profile [25, 26]. Here, we particularly concern about the regulatory connection changes between disease and normal states. So we apply CMI-PC method to reconstruct the disease and normal GRNs [27–29]. Mutual information (MI) is a measure of the mutual dependence between the two variables. It is increasingly popular in GRN reconstruction for the ability to measure non-linear dependency [30, 31]. Conditional mutual information (CMI) in gene pairs is the expected value of the mutual information of two interest genes given the joint regulation by other genes [32]. MI is a special case of zero-order CMI. The MI of variables *X* and *Y*, CMI of variables *X* and* Y* given *Z* are calculated by a widely-used estimation method [33] as

1 $$I(X,Y) = \log \sum\limits_{i,j} {\log } \frac{p(i,j)}{{p_{x}(i) p_{y} (j)}}$$

2 $$I(X,Y|Z) = \sum\limits_{i,j,k} {\mathop p\nolimits_{x,y,z} (i,j,k)} \log \frac{{p_{z} (k)p_{x,y,z} (i,j,k)}}{{p_{x,z} (i,k)p_{y,z} (j,k)}}$$

The approach partitions the supports of *X*, *Y*, *Z* into bins with finite size, where the marginal, joint, and conditional probability mass functions are denoted by *p* with the appropriate subscripts. $p_{x\left( i \right)} = \mathop \smallint \limits_{i}^{ } dx\mu_{x} \left( x \right), p_{y\left( i \right)} = \mathop \smallint \limits_{i}^{ } dy\mu_{y} \left( y \right), p\left( {i,j} \right) = \mathop \smallint \limits_{i}^{ } \mathop \smallint \limits_{j}^{ } dxdy\mu \left( {x,y} \right)$, and $\mathop \smallint \limits_{i}^{ }$ means the integral over the bin *i*.

Similar to MI, a higher CMI value indicates a closer relationship between the variables *X* and *Y* given variable(s) *Z*. Path consistency (PC) algorithm is used to remove the edges from the background network based on CMI values. The process is, for an adjacent gene pair *X* and *Y*, first, calculate MI (0-order CMI). If the value is low or zero, delete the edge between them. Next, select the adjacent gene *Z* of them and compute first-order CMI *I(X,Y|Z*) and repeat the step to delete edges that are independent or not strongly connected until no edge that can be deleted. The procedure will continue until there is no higher order CMI. The threshold values for deletion are the same in the two different conditions.

In this way, we obtain two specific GRNs in disease and normal samples respectively. The different interaction between genes shows the rewiring raised by the disease effects. We extract the rewiring parts and construct a D-GRN. In detail, we find the same genes with different connections and add their adjacent genes. Then we connect them based on the normal and disease GRNs.

### Community detection

The communities in D-GRN are imperative in the understanding of the functional module about the difference between normal and disease conditions. We apply a fast greedy detection algorithm [34] in the D-GRN to identify the closely-connected gene modules. This algorithm can be briefly described as follows: assuming every independent node in the network is a module. And then it merges modules to make the evaluation standard Modularity (*Q*) increase most until all nodes are involved in one module. Finally, a tree graph will appear with leaves representing gene nodes. Modules can be divided by different tree levels. The most reliable dividing corresponds to the maximum modularity. Modularity (*Q*) can be described as:

3 $$Q = \sum\limits_{i} \left({e{}_{ii} - a_{i}^{2} }\right)$$

4 $$a_{i} = \sum\limits_{j} {e_{ij} }$$

where *e*_*ij*_ is the ratio of numbers of edges connected module *i* and module *j* to total edges.

### Biomarker discovery based on LR-RFE

Biomarkers should be able to effectively distinguish disease from normal samples [17, 35]. The detected network-based gene communities provide a pool of module biomarker candidates. To select better biomarkers in each module, we employ RFE with cross-validations based on logistic regression [36] classifier. Compared to other machine learning methods, LR is easier to implement, interpret, and also is a very efficient classification algorithm [37]. Because of its mathematical interpretability, it has a wide range of applications in the field of biomedicine [38]. The logistic regression can be considered as follows

5 $$\pi_{{\text{i}}} = Pr\left( {\left. {y_{i} } \right|x_{{\text{i}}} ;\theta } \right) = f\left( {x_{i}^{T} } \right) = \frac{{\exp \left( {x_{i}^{T} \theta } \right)}}{{1 + \exp \left( {x_{i}^{T} \theta } \right)}},\quad i = 1,2, \ldots ,n.$$

where $X_{i} = (x_{i1} ,x_{i2} , \ldots ,x_{ip} )^{T} ,$ denotes the p-dimensional gene expression vector. *y*_*i*_ is a corresponding binary variable. *θ* is the vector of the coefficients.

For over-fitting problem, we choose *L*_2_ regularization techniques to avoid, which is defined as

6 $$P\left( {\theta ;\lambda } \right) = \lambda \mathop \sum \limits_{j = 1}^{p} \theta_{j}^{2}$$

where *λ* is a positive tuning parameter used to balance the loss term and penalty term.

RFE law is in the process of continuously training the model [39]. Each time the training is completed, the specified number of low-importance features are deleted. Then new features are trained again. The importance of features is obtained again, and unimportant characteristics are deleted until the number of characteristics meets the predefined settings. In this paper, we delete one gene each time and through cross-validation to find the optimal number of features. If reducing the features will cause a performance loss, then no features will be removed. The selected biomarkers are further verified in the validation datasets.

## Data Availability

The results published here are based in part upon data generated by the TCGA Research Network (http://cancergenome.nih.gov/).

## Abbreviations

GRN: Gene regulatory network
D-GRN: Differential gene regulatory network
CMI-PC: Conditional mutual information-based path consistency
LR-RFE: Logistic regression with recursive feature elimination
PCC: Pearson’s correlation coefficient
ROC: Receiver operating characteristic
AUC: Area under the ROC curve
NOA: Network ontology analysis
GO: Gene ontology
BRCA: Breast invasive carcinoma
MI: Mutual information
CMI: Conditional mutual information
PC: Path consistency
Q: Modularity
